# Preliminary evaluation of near infrared spectroscopy as a method to detect plasma leakage in children with dengue hemorrhagic fever

**DOI:** 10.1186/1471-2334-14-396

**Published:** 2014-07-17

**Authors:** Babs Soller, Anon Srikiatkachorn, Fengmei Zou, Alan L Rothman, In-Kyu Yoon, Robert V Gibbons, Siripen Kalayanarooj, Stephen J Thomas, Sharone Green

**Affiliations:** 1 Reflectance Medical, Inc., 116 Flanders Rd, Suite 1000, Westborough, MA 01581, USA; 2 Department of Anesthesiology, University of Massachusetts Medical School, 55 Lake Avenue, North, Worcester, MA 01655, USA; 3 Division of Infectious Diseases and Immunology, University of Massachusetts Medical School, 55 Lake Avenue, North, Worcester, MA 01655, USA; 4 Department of Virology, Armed Forces Research Institute of Medical Sciences, 315/6 Rajvithi Road, Bangkok 10400, Thailand; 5 Institute for Immunology and Informatics, University of Rhode Island, 80 Washington Street, Providence, RI 02903, USA; 6 Queen Sirikit National Institute of Child Health, 520/8 Rajvithi Road, Bangkok 10400, Thailand; 7 Viral Diseases Branch, Walter Reed Army Institute of Research, 503 Robert Grant Avenue, Silver Spring, MD 20910, USA

## Abstract

**Background:**

Dengue viral infections are prevalent in the tropical and sub-tropical regions of the world, resulting in substantial morbidity and mortality. Clinical manifestations range from a self-limited fever to a potential life-threatening plasma leakage syndrome (dengue hemorrhagic fever). The objective of this study was to assess the utility of near infrared spectroscopy (NIRS) measurements of muscle oxygen saturation (SmO_2_) as a possible continuous measure to detect plasma leakage in children with dengue.

**Methods:**

Children ages 6 months to 15 years of age admitted with suspected dengue were enrolled from the dengue ward at Queen Sirikit National Institute for Child Health. Children were monitored daily until discharge. NIRS data were collected continuously using a prototype CareGuide Oximeter 1100 with sensors placed on the deltoid or thigh. Daily ultrasound of the chest and a right lateral decubitus chest x-ray the day after defervescence were performed to detect and quantitate plasma leakage in the pleural cavity.

**Results:**

NIRS data were obtained from 19 children with laboratory-confirmed dengue. Average minimum SmO_2_ decreased for all subjects prior to defervescence. Average minimum SmO_2_ subsequently increased in children with no ultrasound evidence of pleural effusion but remained low in children with pleural effusion following defervescence. Average minimum SmO_2_ was inversely correlated with pleural space fluid volume. ROC analysis revealed a cut-off value for SmO_2_ which yielded high specificity and sensitivity.

**Conclusions:**

SmO_2_ measured using NIRS may be a useful guide for real-time and non-invasive identification of plasma leakage in children with dengue. Further investigation of the utility of NIRS measurements for prediction and management of severe dengue syndromes is warranted.

## Background

There are approximately 390 million dengue virus (DENV) infections worldwide, resulting in 500,000 hospitalizations and 20,000 deaths annually [1]. The mosquito-borne DENV, of which there are four serotypes, causes substantial morbidity and mortality in tropical and sub-tropical regions of the world [2]. The clinical manifestations of DENV infection range from a self-limited febrile illness comprising fever, rash, headache and myalgias known as dengue fever (DF) to a potentially life-threatening plasma leakage syndrome, dengue hemorrhagic fever (DHF). In the evaluation of DHF patients, plasma leakage may manifest as pleural effusion, ascites, or hemoconcentration [2]. Pleural effusions are a common sign of plasma leakage in patients with DHF and have been positively correlated with disease severity [2-4]. If detected early, the effects of plasma leakage are manageable with judicious use of intravenous fluids; case-fatality rates at experienced medical facilities ranges between 0.5% - 1%. However, progression to DHF often relies on serial history and physical examinations and clinical laboratory evaluations to detect hemoconcentration (rising hematocrit) and thrombocytopenia. Chest x-ray and ultrasound have also been shown to be effective in identification of pleural effusions and/or ascites in DHF patients [3]. While non-invasive, these measurements are not practical for prediction of plasma leakage in resource poor settings. Due to the relatively rapid nature of the onset of shock, these crude measures may not be done at the appropriate time to support preventive fluid administration.

Near infrared spectroscopy (NIRS) is a non-invasive technique which can be used to continuously assess tissue oxygenation in cerebral as well as in muscle tissue. It has been widely used on children [5] and has been shown to have important uses in trauma and critical care settings [6]. Muscle oxygen saturation (SmO_2_) is distinct from tissue oxygen saturation (StO_2_) in that the determination of oxygenated and deoxygenated hemoglobin is performed on spectra which have been corrected for spectral interferences from skin pigment and fat [7]. SmO_2_ has been shown to be sensitive for the early detection of hypovolemia by tracking increased oxygen extraction resulting from peripheral vasoconstriction to shunt blood to the heart and brain [8-10]. Since decreased peripheral perfusion is a clinical observation in patients with DHF [2], we hypothesized that NIRS-determined SmO_2_ would be effective in identifying DHF patients with significant plasma leakage. An easy, non-invasive method which would permit early (i.e. prior to more tradition clinical tools) identification of plasma leakage would be an invaluable tool for early identification of progression to DHF and support fluid management to prevent shock in children with severe dengue.

## Methods

### Study enrollment

Children aged ≥ 6 months and ≤ 18 years of age with suspected dengue virus infection, temperature ≥38.5°C within 24 hours of enrollment and no chronic underlying medical condition were enrolled from the in-patient dengue ward at the Queen Sirikit National Institute of Child Health. Subjects were monitored daily until discharge. Fluid administration and other clinical management were guided by the treating physician according to WHO guidelines [2]. Disease severity was assigned according to the 1997 WHO guidelines. Dengue fever (DF) was defined as confirmed dengue virus infection in the absence of evidence of plasma leakage. Dengue hemorrhagic fever (DHF) was defined as plasma leakage (hemoconcentration ≥ 20% over baseline, pleural effusion, or ascites) in the presence of thrombocytopenia (platelet count ≤ 100,000 mm^3^). Temperature was measured at least every 6 hours. The day of defervescence, designated fever day 0, was the day in which the temperature fell and remained below 38°C with one and two days prior designated as fever days -1 and -2 etc. and after defervescence fever days +1, +2, etc. Blood was collected daily for hematocrit determination. Fever time zero was recorded as the first temperature recording below 38°C on fever day 0. Dengue virus infection was confirmed by RT-PCR and/or dengue serology as previously described [11,12]. This study was approved by the Institutional Review Boards of the Queen Sirikit National Institute for Child Health, the Thai Ministry of Public Health, and the US Army Surgeon General. Written informed consent was obtained from the parent or guardian. Assent forms were obtained from subjects who were at least seven years old.

### NIRS data collection

NIRS data were collected with a prototype version of the CareGuide Oximeter 1100 (Reflectance Medical Inc., Westborough, MA, USA). The CareGuide Oximeter uses two different source-detector separation distances to correct the spectra for interference from skin and fat. Incident light is produced by a broad band NIR LED light source which illuminates the tissue over the range of 700 nm – 900 nm. The sensor has a chip-scale spectrophotometer parallel to a row of light sources spaced at different distances (6 mm to 45 mm) from the spectrometer. One light source, placed 6 mm from the spectrometer, collects light reflected from near the tissue surface, capturing the spectrum associated with the skin pigment. A series of six light sources spaced 5 mm apart and 20 mm-45 mm from the spectrometer are used to illuminate the muscle. The sensor automatically selects the light source which is at an appropriate distance to capture light reflected back from the muscle. Thicker fat layers require longer separations to increase the depth of penetration into the tissue. The muscle spectra are corrected by mathematically removing the spectral information from the skin pigment and SmO_2_ is calculated from the isolated muscle spectrum [13]. Additional details on the device, it’s validation against venous blood, and performance on healthy adults have been previously published [14].

**Figure 1:**
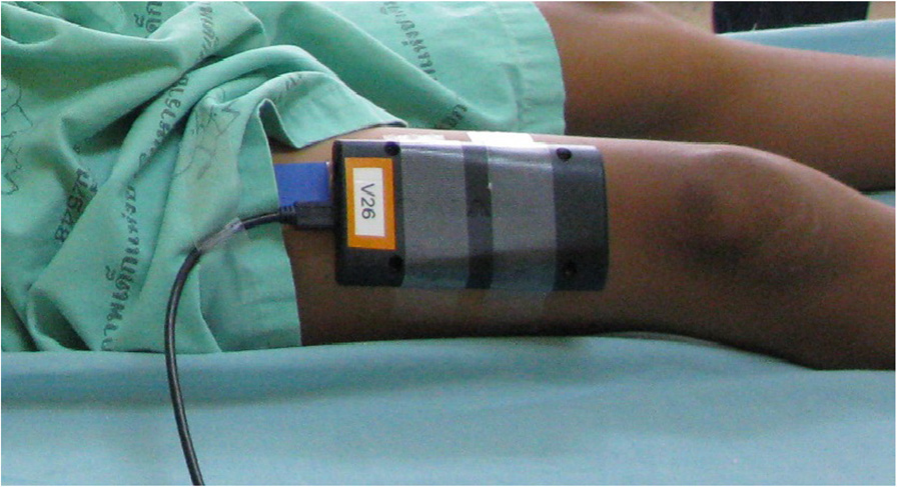
Photograph of prototype CareGuide sensor applied to child’s thigh.

The NIRS sensor was adhered to the deltoid or the thigh with a NIR-transparent double sided adhesive tape (Figure 1). The sensor is attached by a cable to a 10” touch screen computer that is mounted at the bedside. The location of the sensor was at the discretion of the study nurse. The sensor was applied as soon as possible after the child was admitted to the dengue ward. Data was collected every 5 minutes and a minimum average SmO_2_ was calculated for each 30 minute interval using fever time zero as the central reference point. Monitoring was stopped each day to bathe the patient and the sensor was then reapplied. If the sensor came off at night, it was not reapplied until the next morning. Data collection ceased when the child was discharged.

### Ultrasound and radiographic data collection

Ultrasonography of the chest and abdomen was performed by a radiologist daily to assess for pleural effusions. Three sites on the chest were assessed as previously described [3]. Briefly, ultrasound examinations were performed daily with a portable ultrasound scanner (GE Logiq Book) using a 2.5 MHz convex transducer. Standard protocols were used to examine 3 chest sites for fluid accumulation using mid-axillary-longitudinal, a mid-clavicular-longitudinal, and right upper quadrant-transverse views. The presence or absence of fluid in these images was noted by a radiologist who was blinded to the clinical data. If any of the 3 views was positive for fluid, the patient was considered to have pleural effusions on that day.

One day after defervescence (fever day +1), a right lateral decubitus chest x-ray was obtained. A pleural effusion index (PEI) was calculated from the maximal height of pleural fluid/width of hemithorax × 100 [3].

### Data analysis

The minimum average 30-minute SmO_2_ was determined for each study day and a receiver operator characteristic curve (ROC) was constructed to determine the sensitivity and specificity of SmO_2_ to detect pleural effusions observed with the daily ultrasound measurements. The x-ray determined PEI was compared to the minimum SmO_2_ for the same day using linear regression.

## Results

### Study population

**Figure 2:**
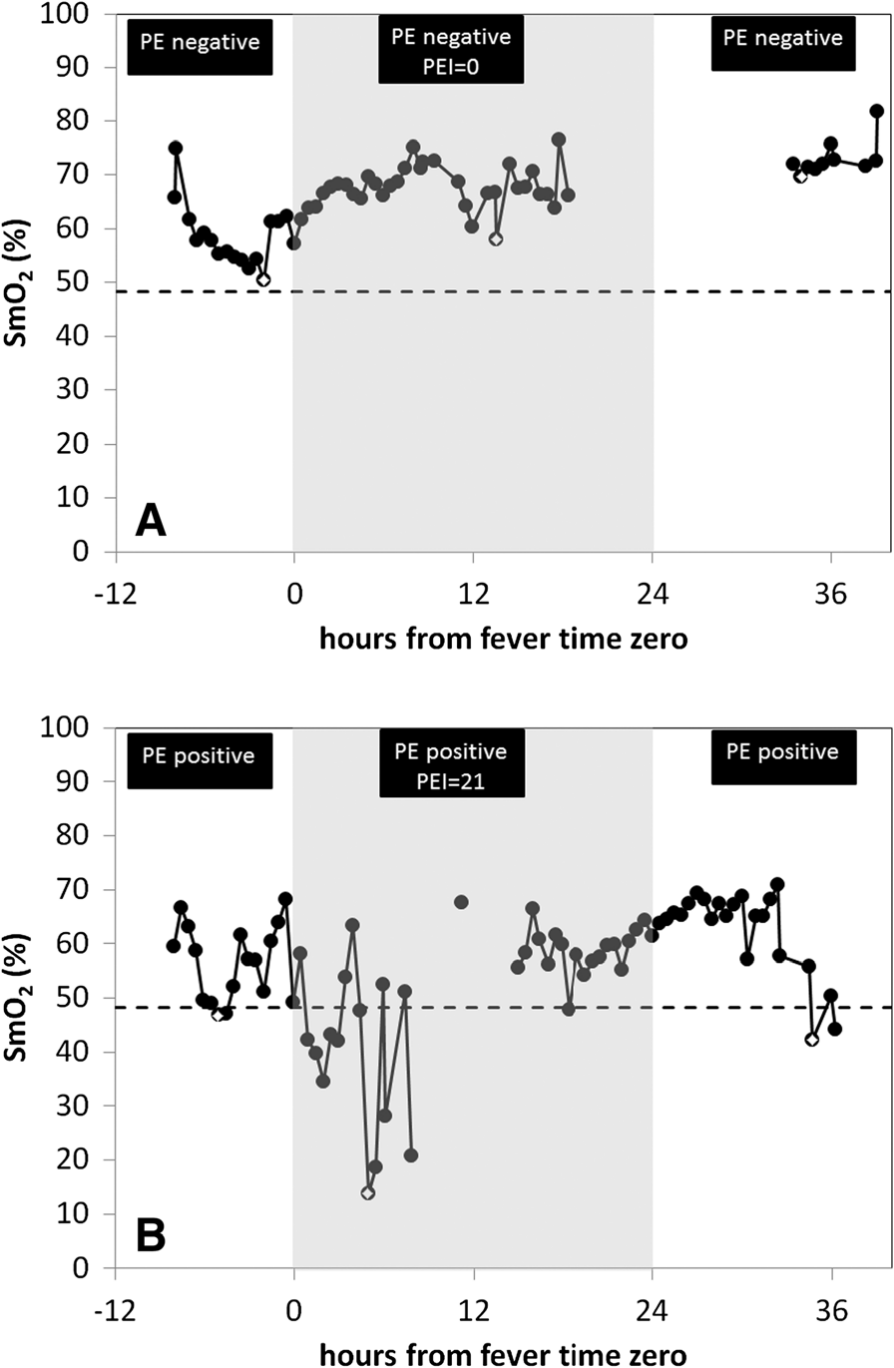
**Representative plots of minimum average 30 minute SmO**_**2 **_**for (A) a subject with no pleural effusion detected and (B) a subject with pleural effusion detected by ultrasound and chest x-ray.** Fever time zero denotes the time of defervescence (temperature ≤38°C). PE = pleural effusion as measured by ultrasound; PEI = pleural effusion index as measured on right lateral decubitus chest x-ray.

NIR spectra were successfully collected for 19 (13 M/6 F) patients intermittently over the enrollment period (range: 2 to 5 days). Patient characteristics at study enrollment are shown in Table 1. The average patient age was 10.2 ± 2.6 years. Three of 19 subjects (16%) progressed to shock. Twelve subjects had the sensor placed on the arm and 7 subjects had data collected on the thigh. There was no association between gender and sensor location, however the thigh placement was used on the younger subjects, who generally had arms too small to accept the sensor. Representative 30 minute average SmO_2_ for two subjects, one without and one with evidence of pleural effusion, collected during the ‘critical period’ from fever day – 1 through fever day +1 are shown in Figure 2.

**Table 1:** Baseline characteristics of subjects at study entry

**Clinical diagnosis at discharge**	**DF**	**DHF**
Number of subjects	8	11
Age (mean ± SE)	10.5 ± 0.3	10.0 ± 0.2
Sex (male/female)	6/2	7/4
Hematocrit (%) (mean ± SE)	39.8 ± 0.3	42.3 ± 0.6
AST (mean ± SE)	70 ± 11	102 ± 16
Presence/Absence of pleural effusion (by ultrasound)	0/8	2/7*
Days ill prior to enrollment (mean ± SE)	4.5 ± 0.1	4.3 ± 0.1
Fever day (mean ± SE)	-0.25 ± 0.1	-0.73 ± 0.1

* Data not available for 2 subjects.

### Association of SmO2 with pleural effusion

**Figure 3:**
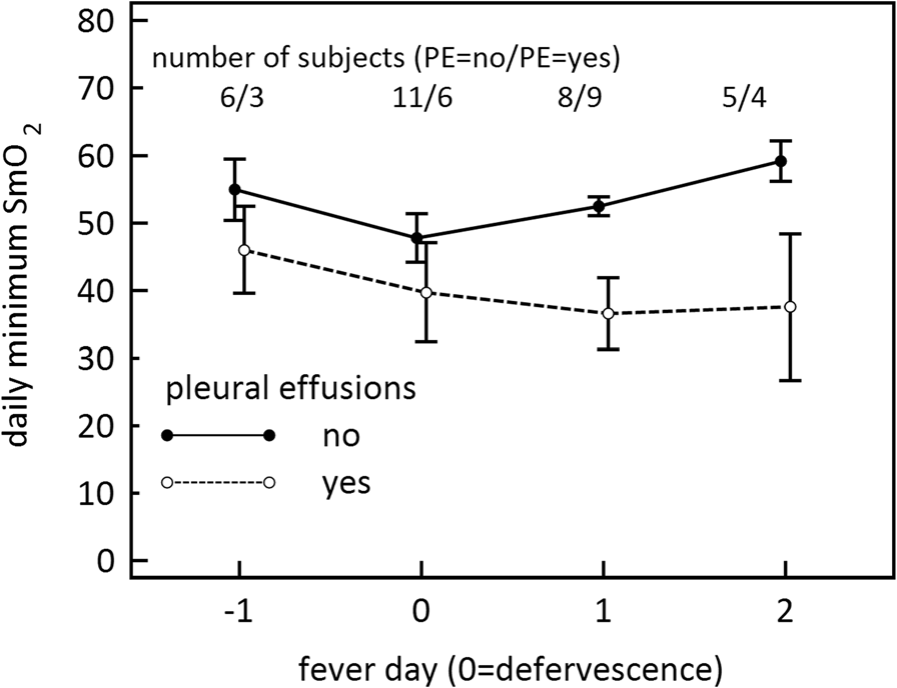
**Minimum daily SmO**_**2 **_**by fever day for patients without pleural effusions (closed circles) and with pleural effusions (open circles) determined from ultrasound.** Fever day = 0 is the day of defervescence. Mean ± standard error.

**Figure 4:**
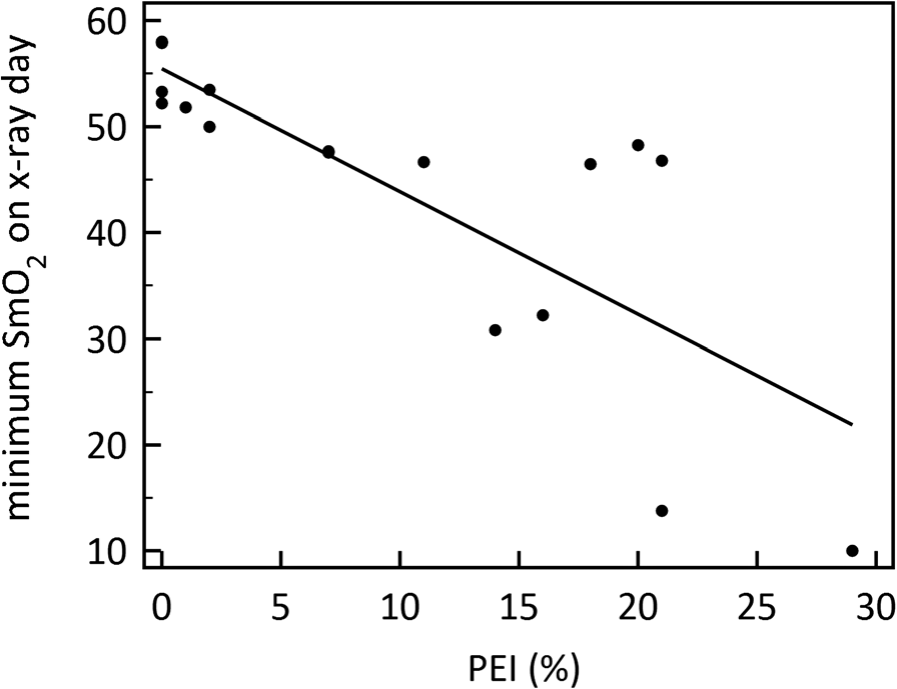
**Linear regression of minimum SmO**_**2 **_**with pleural effusion index (PEI) determined from a chest x-ray taken on the same day; R**^**2**^ **= 0.61.**

Maximal plasma leakage and shock in dengue typically occurs within 24 hours of defervescence. To compare all subjects at similar times during their disease progression, SmO_2_ was plotted by fever day rather than study day, where fever day = 0 represents the day of defervescence (Figure 3). Between fever day -1 and fever day 0, average minimum SmO_2_ decreased for all patients; however on fever day +1, on average, SmO_2_ increased for patients without ultrasound evidence of pleural effusion, and conversely decreased for those patients with evidence of pleural effusion. Figure 4 shows the relationship between minimum SmO_2_ on fever day +1 and the PEI determined from chest x-ray on that day. We found an inverse linear relationship between the two parameters (R^2^ = 0.61) with low values of SmO_2_ corresponding to a high pleural effusion index.

**Figure 5:**
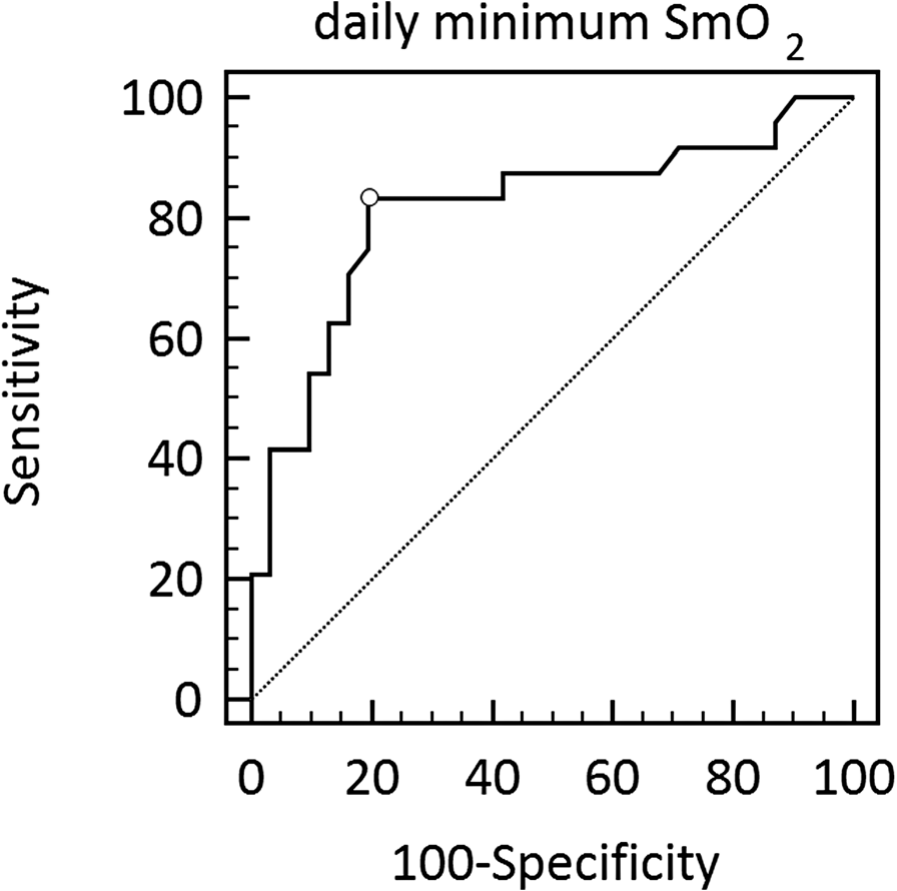
**Receiver operator characteristic curve for daily minimum SmO**_**2 **_**as an indicator of pleural effusion.** At the SmO_2_ cut-off value of 48.3%, marked with an open circle, the sensitivity is 83% and the specificity is 81%.

Figure 5 shows the ROC curve for minimum SmO_2_ as an indication of pleural effusion detected with ultrasound on each study day (55 data points). At the SmO_2_ cut-off value of 48.3% the sensitivity is 83% and the specificity is 81%. The area under the ROC curve is 0.81 (95% CI: 0.69 – 0.91).

## Discussion

To our knowledge, this is the first study to use NIRS to evaluate dengue patients for markers of plasma leakage that may result in shock. Our data show that SmO_2_ values less than 48% have a high sensitivity and specificity for being associated with pleural effusions in the study patients. A tool which permits real time detection of plasma leakage via SmO_2_ monitoring may help to determine which individuals will require earlier fluid intervention, thereby reducing the number of dengue patients who proceed to shock.

Earlier studies using strain gauge plethysmography demonstrated that pleural effusion in dengue patients was accompanied by microvascular fluid leakage into the extravascular space [15]. Recent studies confirm that pleural effusions were the most common sign of plasma leakage in DHF patients [3,4]. Plasma leakage may result in reduced intravascular volume, a higher risk for shock and thereby reduced SmO_2_. It has been previously demonstrated that SmO_2_ is a very early indicator of central hypovolemia using a human laboratory model of pre-shock hemorrhage: lower body negative pressure (LBNP). Significant decreases in SmO_2_ occurred well before decreases in blood pressure and arterial oxygen saturation by pulse oximetry (SpO_2_) and increases in heart rate [8,9]. In the LBNP model, decreases in SmO_2_ were highly correlated with increases in total peripheral resistance, indicating that this observed increase in oxygen extraction results from vasoconstriction that is shunting blood away from the skeletal muscle toward the heart in an effort to maintain normal blood pressure [8,10]. In a similar fashion, the decreases in SmO_2_ observed in these DHF patients likely result from intravascular volume depletion and subsequent compensatory vasoconstriction.

In a study by Libraty et al., the ratio of extracellular to intracellular water (ECW/ICW) was determined by bioelectrical impedance spectroscopy for patients with DF, DHF and other febrile illnesses [16]. Around the time of defervescence, the ECW/ICW ratio increased in proportion to disease severity. The authors suggested that the positive fluid balance that accompanied the increase in extracellular water was due to a reduction in renal water clearance [16]. An increase in extravascular water could reduce tissue blood flow and increase oxygen extraction, thereby lowering SmO_2_. During the time surrounding defervescence and fluid administration, it is not known if the decrease in SmO_2_ is a result of hypovolemia or excess interstitial water reducing blood flow. Gaining a better understanding of this may help in developing tools to guide fluid resuscitation for dengue patients in a manner which will avoid fluid overload.

One limitation of this study may be the use of daily minimum SmO_2_ for statistical analysis. Patients without pleural effusion had fewer NIRS readings per day than patients with pleural effusion, likely because these patients were less sick and therefore more ambulatory.

This pilot study investigated the noninvasive monitoring of SmO_2_ in patients with dengue infection. Low levels of SmO_2_ were shown to be associated with pleural effusions detected with ultrasound and chest x-ray. This technology provides continuous SmO2 readout to track of the progression and treatment of patients with diseases which cause plasma leakage and heightened risk of shock. This initial study determined a SmO2 cutoff of 48%, but this value would need to be confirmed in additional dengue cohorts of both adults and children. Once validated, at risk patients could be routinely monitored because the display provides immediate feedback to the clinician. The SmO_2_ monitor is significantly lower in cost than ultrasound equipment, but it is recognized that to be widely adopted in countries with limited resources the cost to use it must be modest.

## Conclusions

A pilot study of Thai children hospitalized with acute dengue virus infection demonstrated that minimal daily SmO_2_ as measured by NIRS was found to correlate with pleural effusions in hospitalized children with acute dengue virus infection. ROC analysis revealed a cut-off value for SmO_2_ (48%) which yielded high specificity and sensitivity. Further studies to further define and validate the utility of NIRS monitoring to detect plasma leakage in dengue are warranted.

## Competing interests

BS and FZ are employees of Reflectance Medical Inc. and hold stock or stock options in the company. BS is an officer of Reflectance Medical. The remaining authors have no conflicts to declare.

## Authors’ contributions

Conceived and designed the experiments: ALR BS IY SJT SG. Performed the experiments: RVG AS SJT SK. Analyzed the data: BS FZ ALR AS SJT SG. Enrolled patients: AS SK. Wrote the paper: BS AS ALR IY RVG SK SJT SG. All authors read and approved the final manuscript.

## Pre-publication history

The pre-publication history for this paper can be accessed here:

http://www.biomedcentral.com/1471-2334/14/396/prepub
